# How Does the Addition of Kollidon^®^VA64 Inhibit the Recrystallization and Improve Ezetimibe Dissolution from Amorphous Solid Dispersions?

**DOI:** 10.3390/pharmaceutics13020147

**Published:** 2021-01-23

**Authors:** Joanna Szafraniec-Szczęsny, Agata Antosik-Rogóż, Mateusz Kurek, Karolina Gawlak, Anna Górska, Sebastian Peralta, Justyna Knapik-Kowalczuk, Daniel Kramarczyk, Marian Paluch, Renata Jachowicz

**Affiliations:** 1 Department of Pharmaceutical Technology and Biopharmaceutics, Faculty of Pharmacy, Jagiellonian University Medical College, Medyczna 9, 30-688 Krakow, Poland; agata.antosik@uj.edu.pl (A.A.-R.); mateusz.kurek@uj.edu.pl (M.K.); an.gorska@uj.edu.pl (A.G.); renata.jachowicz@uj.edu.pl (R.J.); 2 Department of Physical Chemistry and Electrochemistry, Faculty of Chemistry, Jagiellonian University, Gronostajowa 2, 30-387 Krakow, Poland; gawlak@chemia.uj.edu.pl; 3 Pharmacy and Pharmaceutical Technology Department, School of Pharmacy, University of Granada, Campus de Cartuja s/n., 18071 Granada, Spain; seperalta@ugr.es; 4 Faculty of Science and Technology, Institute of Physics and SMCEBI, University of Silesia, 75 Pułku Piechoty 1a, 41-500 Chorzów, Poland; justyna.knapik-kowalczuk@smcebi.edu.pl (J.K.-K.); daniel.kramarczyk@smcebi.edu.pl (D.K.); marian.paluch@us.edu.pl (M.P.)

**Keywords:** ezetimibe, solid dispersion, amorphization, dissolution, contact angle

## Abstract

Amorphization serves as a strategy for the improvement of poor dissolution characteristics of many drug compounds. However, in many formulations the content of polymeric stabilizer is high, which is undesirable from the perspective of future applications. Thus, studying the composition-dependent stability of amorphous solid dispersions seems to be demanded. In this paper, we describe the amorphization of ezetimibe, a lipid-lowering drug, in the spray drying process and investigate the effect of polyvinylpyrrolidone-*co*-poly(vinyl acetate) (PVP/VA) content on the physical stability and dissolution characteristics of the drug. Fully amorphous systems were obtained when the concentration of the polymer in solid dispersion was as low as 20%. The amorphization led to the dissolution enhancement by even 70%, with a noticeable sudden increase at around 40% of PVP/VA content and very small variations for systems having 66–90% PVP/VA. It was also correlated to wettability characteristics of solid dispersions, which may suggest that in the vicinity of 40% of the polymer content, the behavior of the system becomes independent of the PVP/VA content.

## 1. Introduction

Over the past decade, cardiovascular diseases (CVDs) have accounted for over 30% of all global deaths, making it the main cause of death worldwide [1]. A major proportion of morbidity statistics is related to heart attacks and strokes, which result from the deposition of fatty material and cholesterol in the lumen of blood vessels [2]. The pathogenesis of so-called atherosclerotic CVD is related to serum cholesterol and its lipoprotein carriers, mainly low-density lipoprotein (LDL). Given that the reduction of LDL level lowers the risk of CVD, it is the first-line strategy established in the treatment of dyslipidemia. Randomized trials have shown that a 1% reduction in LDL level reduces the risk of atherosclerotic CVD by approximately 1% in high-risk patients [3].

The current mainstay of primary and secondary prevention of cardiovascular diseases are statins, HMG-CoA reductase inhibitors [4]. However, many patients cannot achieve an adequate level of LDL from being treated only with statins and require a greater modification in lipid profile. They are treated with a second agent, ezetimibe (EZT), which is the first lipid-lowering drug that inhibits the uptake of dietary and biliary cholesterol from the small intestine and does not affect the plasma level of drugs typically used in patients with hypercholesterolemia [5,6]. The mechanism of its action involves the cholesterol transport protein Niemann-Pick C1-Like 1 (NPC1L1). Ezetimibe is the first drug not affecting the absorption of fat-soluble vitamins, triglycerides, or bile acids [7]. It is weakly acidic in nature (pKa ≈ 9.48) and virtually insoluble in water. Although EZT contains ionizable functional groups, its solubility exhibits no dependency on pH range characteristic for the gastrointestinal tract. However, it exhibits good membrane permeability (logP = 4.5) and belongs to class II of the Biopharmaceutics Classification System (BCS) [8]. Given its extremely low solubility in water, the absolute bioavailability cannot be determined [5]. However, the hydrophobic character of the drug indicates that the bioavailability is low.

This property is a common obstacle in drug development; hence, several strategies have been introduced to enhance ezetimibe characteristics. They include the formation of solid dispersions utilizing melting, solvent evaporation, supercritical carbon dioxide technology, and crystal engineering, with the generation of nanocrystals and cocrystals [9,10,11,12,13,14]. Crystal engineering has attracted considerable attention of formulation scientists, mainly due to the high thermodynamic stability of crystalline substances. However, almost 80% of active pharmaceutical ingredients (APIs) exhibit polymorphism and undergo solid-state phase transitions upon various stimuli, which may alter drug solubility and dissolution. On the other hand, the amorphization of API leads to a great enhancement of drug dissolution, but thermodynamic instability of high energy solids acts as a serious limitation, as uncontrolled recrystallization during storage or after administration can cause severe damage to the body. Such systems require using stabilizers, which can inhibit the restoration of the drug crystal structure and improve its solubility performance.

The enhancement of dissolution using solid dispersions is a result of various effects, including an increase in particle surface area associated with particle size reduction, solubilization of molecularly dispersed drug by polymer matrix-forming molecules, increase in wettability, and changes within the crystalline structure of drug [15]. One of the most intensely investigated fields in recent years is the formation of amorphous solid dispersions with water-soluble polymers. Amorphous solids have attracted particular attention in the pharmaceutical field due to the fact of the high level of supersaturation in solution after oral administration [16], which provides a superior bioavailability in comparison with products containing crystalline APIs. The excess of free energy drives the dissolution; however, it also results in physical instability of molecularly disordered systems manifested by a tendency towards recrystallization [17]. To prevent the reconstruction of the crystal lattice and ensure improved stability of amorphous API over time and under a variety of stress conditions, such as elevated temperature and humidity, drugs are mixed with freely soluble polymers having high glass transition temperature (T_g_) [18].

Studies performed so far have shown that physical stabilization of amorphous solid dispersions can be attributed to several factors such as a reduction in molecular mobility, altering the thermodynamic driving force for crystallization by reducing the chemical potential, and the increase of energy barrier. Regardless of the specific mechanism, the maximum stabilization of the amorphous API requires the components to be intimately mixed at the molecular level [19]. Miscibility is hence defined in terms of a system containing a single supersaturated metastable phase of drug and polymer. Thermodynamic criteria of physical stabilization of amorphous solid dispersions include a sufficiently positive combinatorial entropy and the presence of favorable API–polymer intermolecular interactions such as hydrogen bonding and dipole–dipole interactions [20]. Given that two types of systems can be distinguished, i.e., those in which the two components are miscible at all compositions and those miscible only within certain areas of the phase diagram, the study of composition-dependent stability of amorphous solid dispersions seems to be demanded [21].

The use of pharmaceutically acceptable polymers such as polyvinylpyrrolidone (PVP), polyvinylpyrrolidone-*co*-poly(vinyl acetate) (PVP/VA), polyethylene glycol (PEG), or hydroxypropyl methylcellulose (HPMC) reduces molecular mobility of API molecules and inhibits nucleation and crystal growth, which leads to increased stability of amorphous solids over pharmaceutically relevant time scales [22,23]. Importantly, even a small amount of polymeric crystallization inhibitor can stabilize the amorphous form of numerous API as described for felodipine, carbamazepine, or acetaminophen [24,25,26] due to the fact of strong specific intermolecular interactions such as hydrogen bonds that influence the nucleation and crystal growth.

Spray drying is a solvent-based method for manufacturing amorphous solid dispersions and is appropriate for thermally labile APIs [27,28]. It has been applied for the formation of solid dispersion containing celecoxib [29], tadalafil [30], itraconazole [31], and bicalutamide [32,33].The technique relies on the formation of solid microparticles in a the presence of hot drying gas. It comprises the atomization of the drug-containing liquid, droplet-to-particle conversion, and the separation of obtained particles from the gas using cyclone [34]. The scalability provides the possibility of continuous manufacturing of particles having desirable characteristics and good uniformity of molecular dispersion [35]. This industrial technology was used in the preparation of marketed products such as Intelence^®^ or Incivek^®^ [36].

The work presented herein aims at investigating the effect of various content of vinylpyrrolidone-vinyl acetate copolymer on the physical stability and dissolution characteristics of an anticholesterol agent, namely ezetimibe. Although the research on the mechanisms of amorphous ezetimibe stabilization have been already studied (the effect of molecular mobility), there is a lack of research on the correlation between polymer content, the stability of the amorphous drug, and its dissolution. Solid dispersions were obtained using spray drying and characterized using scanning electron microscopy and laser diffraction to determine how the particle size and morphology changed upon processing and the increasing amount of the polymer. Molecular characteristics of the systems included X-ray diffractometry, differential scanning calorimetry, and infrared spectroscopy. These methods were applied to study the mechanisms involved in the stabilization of disordered ezetimibe. The wettability of the samples was assessed by the measurements of content angles of the samples. The dissolution was determined in a pharmacopoeial paddle apparatus.

## 2. Materials and Methods

### 2.1. Materials

Ezetimibe (EZT,(3R,4S)-1-(4-fluorophenyl)-3-[(3S)-3-(4-fluorophenyl)-3-hydroxy- propyl]-4-(4-hydroxyphenyl)azetidin-2-one, >99.5%, Hangzhou Hyper Chemicals Limited, Zhejiang, China) was used as a model drug. Vinylpyrrolidone-vinyl acetate copolymer (Kollidon^®^VA64, PVP/VA, BASF, Ludwigshafen am Rhein, Germany) was used as an excipient. Sodium lauryl sulfate (SLS, BASF, Ludwigshafen am Rhein, Germany) was used to prepare dissolution medium. Ethanol (absolute, 99.8%, pure p.a., Avantor Performance Materials Poland, Gliwice, Poland) was used as a solvent in spray drying processes. Cyclohexane (ACS, pure p.a., Avantor Performance Materials, Gliwice, Poland) was used as a dispersant in laser diffraction measurements. All chemicals were used as received. Distilled water was used to prepare all aqueous solutions.

### 2.2. Spray Drying (SD)

Solid dispersions containing ezetimibe and PVP/VA (from 5% to 90% wt.) were dried from ethanolic solutions using a Büchi Mini Spray Dryer B-191 (Flawil, Switzerland). The following parameters were maintained: inlet temperature: 76 °C, outlet temperature: 58 °C, aspirator flow: 100%, gas flow rate: 600 L/min, liquid flow rate: 3.4 mL/min, 0.7 mm in diameter nozzle, yields ca. 70%. The samples were further dried under vacuum to remove residual solvent.

### 2.3. Scanning Electron Microscopy (SEM)

Particle size and morphology of solid dispersions were determined using a Phenom Pro desktop electron microscope (PhenomWorld, Thermo Fisher Scientific, Waltham, MA, USA) equipped with a CeB_6_ electron source and a backscattered electron detector. The powder was placed on the conductive adhesive tape previously glued to a specimen mount. The excess sample (loosely bound to the tape) was removed using a stream of nitrogen. The samples were measured using a holder for non-conductive samples at acceleration voltage equal to 10 kV.

### 2.4. Differential Scanning Calorimetry (DSC)

The thermal properties of the investigated mixtures were examined using a Mettler–Toledo DSC 1 STAR^e^ System. The measuring device was equipped with an HSS8 ceramic sensor having 120 thermocouples and a liquid nitrogen cooling station. The instrument was calibrated for temperature and enthalpy using zinc and indium standards. The glass transition temperature was determined as the midpoint of the glass transition step, while the crystallization and melting temperatures were established from the onsets of the exothermic and endothermic peaks, respectively. The samples were measured in an aluminum crucible (40 μL). All measurements were carried out with and without annealing (T = 353 K; t = 5 min). The experiments were performed from 298 to 453 K with a heating rate of 10 K/min.

### 2.5. Powder X-ray Diffraction (PXRD)

The diffraction patterns of the samples were registered using a Rigaku Mini Flex II X-ray diffractometer (Tokyo, Japan) within the angular range 3–70° 2θ scanned in steps of 0.02 with a scan speed equal to 5°/min. The measurements were carried out at ambient temperature using a monochromatic Cu Kα radiation (λ = 1.5418 Å). The samples in form of powder were placed in a standard glass sample holder without milling prior to the measurement.

### 2.6. Laser Diffraction Measurements

The measurements of particle size distribution were performed using a Mastersizer 3000 equipped with a HydroEV unit (Malvern Instruments, Malvern, United Kingdom) in a wet method. Cyclohexane (reflective index, RI = 1.426) was filtered through the G5 sintered disc filter funnel and placed in the beaker. The sample in powder form was added to the dispersant until the obscuration reached the given value (between 5–20%) and then the measurement was carried out. The rotational speed of the mixer was 1500 rpm. The relationship between the particle size and light intensity distribution pattern was found based on the Fraunhofer diffraction theory. The reported data represents the averages from ten series of measurements of each sample and the distribution span.

### 2.7. Fourier Transform Infrared Spectroscopy (FTIR)

A Nicolet iS10 FT-IR spectrometer (Thermo Fisher Scientific, Waltham, MA, USA) equipped with a Smart iTR™ ATR (Attenuated Total Reflectance) sampling accessory with a diamond as an ATR crystal was used to collect the vibrational spectra of powders. Spectra were collected with 4 cm^−1^ resolution within the range 600–4000 cm^−1^. Presented data represent an average of 64 scans for each sample.

### 2.8. Dissolution Study

A pharmacopeial paddle dissolution apparatus Vision Elite 8 (Hanson Research, Chatsworth, CA, USA) equipped with a VisionG2 AutoPlus Autosampler was used to determine the dissolution of ezetimibe. A method recommended by the FDA for EZT tablets (10 mg of EZT, 500 mL of 0.5% SLS, 75 rpm, 37 ± 0.5 °C) was applied. The sink conditions were maintained as the solubility of EZT in 0.5% SLS was determined to be equal to 66.2 ± 0.2 mg/L. The amount of dissolved drug was assayed at 248 nm using a UV-1800 spectrophotometer (Shimadzu, Kioto, Japan) equipped with flow-through cuvettes. The tests were carried out in triplicate, and the presented results represent averages with their standard deviations (Mean ±SD).

### 2.9. Wettability Study

The sessile drop technique was applied to determine contact angles of EZT and EZT-PVP/VA solid dispersions with a DSA255 drop shape analyzer (Krüss, Hamburg, Germany). The droplet of distilled water of volume equal to 2 µL was deposited on the surface of solid dispersions compressed using an Atlas^TM^ manual 15Ton hydraulic press (Specac, Kent, UK) with a load pressure of 1.5 tons that was applied for each sample for 15 s.

## 3. Results

### 3.1. Particle Size and Morphology

**Figure 1:**
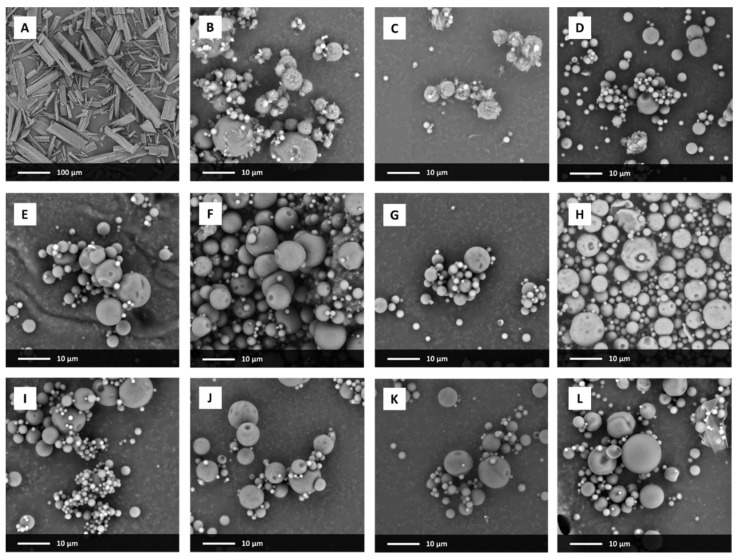
SEM images of raw ezetimibe (**A**), drug spray-dried without a carrier (**B**) and EZT-PVP/VA solid dispersions containing 5% (**C**), 20% (**D**), 25% (**E**), 33% (**F**), 40% (**G**), 50% (**H**), 66% (**I**), 75% (**J**), 80% (**K**) and 91% (**L**) of PVP/VA, respectively.

**Figure 2:**
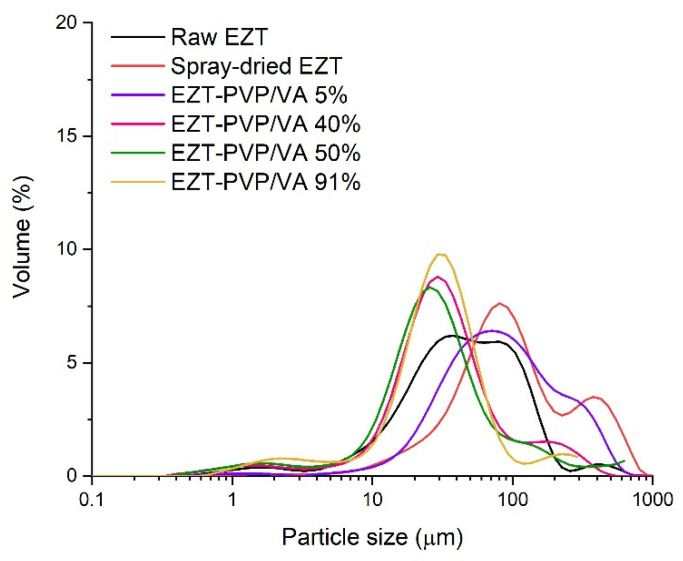
Particle size distribution of raw ezetimibe, drug spray-dried without a carrier and selected EZT-PVP/VA solid dispersions.

The analysis of SEM pictures indicates that the particles of raw ezetimibe exhibited plate-like shape with length ranging between 40 and 300 µm and different width (Figure 1A). The elongated prisms had sharp edges and a smooth surface with a visible transversal. Particle size distribution determined using a laser diffraction technique was wide, without well-resolved maximum (Figure 2). It exhibited a tail corresponding to the presence of long particles (seen in the SEM picture). The differences between SEM and laser diffraction data resulted from the differences in the used methodology [37]. In SEM, the size parameter is measured as the actual size of the visualized object. In the laser diffraction method, the size of the object is calculated based on the analysis of the angular scattering intensity of the laser beam passing through a dispersed sample. The software calculates the sizes of the particles based on the Mie or Fraunhofer diffraction theories and approximates them by the equivalent volume sphere; i.e., the size of the analyzed particle is equal to a diameter of a sphere with the same volume as the particle in the sample. Thus, the obtained data represent the volume percentage of particles having a certain size.

Spray drying of the API (without the polymer) led to the morphological changes of the particles. They were spherical, with a diameter ranging between several hundreds of nanometers to 20 µm, and their surface was rugged. Bigger particles were covered with smaller ones, which correlated and explained very high values of particle diameters determined by laser diffraction (Table 1).

After spray drying with PVP/VA, no significant differences were observed for systems containing different amounts of the polymer. All the systems formed spherical particles typical for the applied process. The diameter of the formed particles did not exceed 12 µm and the surface was smooth and homogenous (Figure 1D–L). Particle size distribution was narrower than for the samples not containing polymeric excipient (Figure 2), with the maxima shifted towards lower particle size.

However, in the case of the system containing only 5% of the carrier, the distribution of particle diameters was wide, with the median {D_v_(50)} lying at 78.9 µm (Table 1). It resulted from particle agglomeration and recrystallization of EZT at the surface of particles. As seen in the SEM picture (Figure 1C) the surface of particles is rugged and covered with plate-like crystals.

### 3.2. Molecular Structure and Interactions

**Figure 3:**
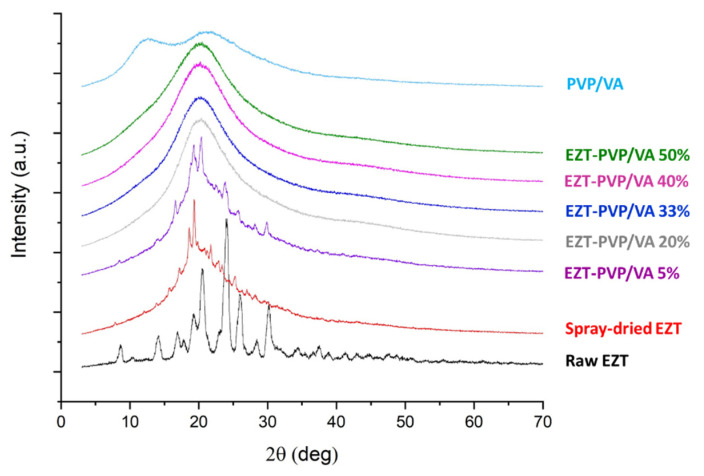
X-ray diffraction patterns of raw and spray-dried ezetimibe, PVP/VA and selected EZT-PVP/VA solid dispersions.

Spray drying led not only to morphological changes in particle size but also to rearrangement in the crystal lattice as confirmed by X-ray powder diffractometry (Figure 3). Sharp Braggs peaks registered for raw ezetimibe correspond to the orthorhombic crystal system described in the Cambridge Crystallographic Data Centre (CCDC) under the deposition number 786811. It belongs to a P 2_1_ 2_1_ 2_1_ space group, with the following cell parameters: *a* 5.94606(19)Å *b* 15.8898(5)Å *c* 21.3765(6)Å, *α* 90° *β* 90° *γ* 90°. Two polymorphs have been reported so far. The positions of Braggs peaks at 8.3°, 13.7°, 20.2°, 23.9°, 25.5°, and 29.8° indicate that the investigated sample is the first crystalline form according to a US patent no WO 2005/009955 A1 [38].

The analysis of the diffractogram collected for ezetimibe spray-dried without the carrier confirms drug amorphization. However, there is a fraction of crystalline material in the sample, which contributes to the diffraction pattern, i.e., Braggs peaks superimposed on the amorphous halo. Similar observations were made regarding the solid dispersion containing 5% of PVP/VA. Although the peak intensity is very low, their position indicates the restoration of the initial crystal form.

Given that amorphous materials do not have a long-range periodic array, they do not have a diffraction pattern. Thus, the lack of Braggs peaks in the diffraction patterns collected for the other systems confirms drug amorphization. It indicates that even 20%-content of PVP/VA sufficiently stabilizes the highly energetic amorphous state of ezetimibe. These results are consistent with the data presented by Knapik et al. [39]. The authors analyzed the role of molecular dynamics in the recrystallization behavior of amorphous ezetimibe. It was concluded that molecular mobility is the main factor responsible for the devitrification of the drug at ambient temperature and pressure; the relaxation occurs within three weeks. However, the addition of 20 wt.% of amphiphilic carrier Soluplus^®^ (a graft copolymer of polyvinyl caprolactam, polyvinyl acetate, and polyethylene glycol) increased the physical stability of a disordered state of the drug for over four years. The formation of the drug-polymer system also led to a 6-fold increase in drug solubility. The same research group also analyzed a co-amorphous formulation of ezetimibe with a low-molecular weight drug, namely-indapamide [40]. In this case, the recrystallization of EZT was significantly inhibited by using a small amount of indapamide, only 8.8 wt.%. The molecular dynamics was slowed down with increasing the content of the stabilizer.

The effect of stabilization of high-energy solids relates to either antiplasticizing activity of the applied polymer or the formation of strong molecular interactions, such as hydrogen bonds, which affect nucleation and crystal growth [41].

**Figure 4:**
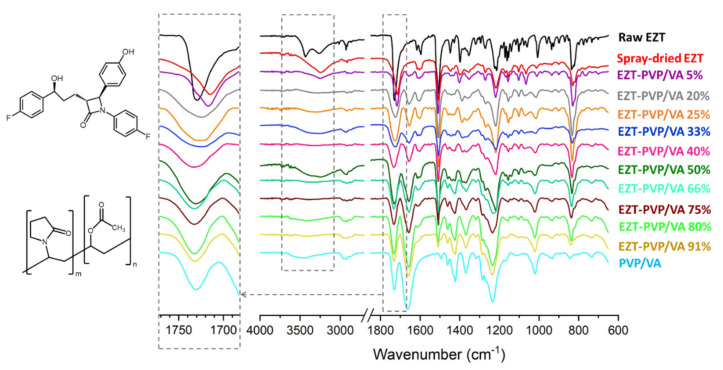
FTIR spectra of raw EZT, PVP/VA and EZT-PVP/VA solid dispersions.

To identify the molecular interactions, we applied infrared spectroscopy. The spectrum of raw ezetimibe is characterized by intense infrared absorption peaks corresponding to stretching vibrations of functional groups (Figure 4). The broad peak at 3200–3600 cm^−1^ is a hydroxyl stretching mode, and the peaks at 1729 cm^−1^ and 1598 cm^−1^ correspond to carbonyl group vibration of the azetidinone ring and C-C vibrations of the aromatic rings, respectively. The C-F stretching vibrations of fluorophenyl rings are observed in the vicinity of 1150 cm^−1^, and the *para*-substituted benzene rings vibration at around 827 cm^−1^.

The shift in the peak position is visible while comparing crystalline and amorphous ezetimibe. In the crystal, the stretching vibration of the hydroxyl group forms a broad band with two distinct maxima, at 3440 cm^−1^ and 3268 cm^−1^, while in the amorphous sample the band is broader and exhibits a single maximum at 3246 cm^−1^. It results from the lack of long-range order as the molecules can occupy numerous positions, adopt various conformations, and interact with neighboring molecules at different locations, thus exhibiting a larger distribution of the absorption frequencies. The presence of two maxima in the crystal sample indicates the presence of the two populations of interactions, most likely because of the presence of two hydroxyl groups. In the amorphous state, only one type of interaction is observed. Given that it is shifted towards a lower wavenumber, it is stronger than the interaction presents in the crystalline phase [42]. In the solid dispersions, the peak is barely visible.

The other shift is observed for the carbonyl group. It appears at 1729 cm^−1^ in the EZT crystal, while in the molecularly disordered sample, it is shifted towards a lower wavelength (1715 cm^−1^) and develops a distinct long-wavelength shoulder. In the solid dispersion containing 5% of the polymer, this peak appears at 1715 cm^−1^, while the increase in the polymer content leads to the shift towards the position characteristic for PVP/VA (1732 cm^−1^).

Although the presence of hydroxyl groups acting as hydrogen atom donors indicates that ezetimibe can form hydrogen bonds with other molecules, it cannot be demonstrated. No substantial shifts in the peak positions were observed, and the spectra of solid dispersions are a sum of vibrations of individual components of the system. This indicates that the interactions between the drug and the polymer were less favorable than among EZT and PVP/VA themselves. It also suggests that there is another mechanism involved in the stabilization of amorphous ezetimibe.

### 3.3. Thermal Properties

**Figure 5:**
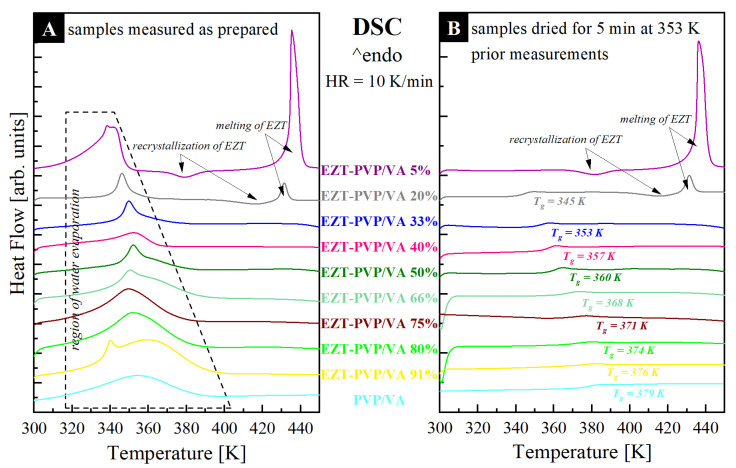
DSC thermograms of EZT-PVP/VA solid dispersions that were measured (**A**) as prepared and (**B**) before drying procedure performed for 5 min at 353 K.

Considering that there are no significant interactions between EZT and PVP/VA, the most probable is that the observed improvement in the physical stability of the amorphous API is related to an antiplasticization effect exerted by the polymer. In this case, polymeric excipient, having a high glass transition temperature (*T_g_*), should raise the *T_g_* of the mixture, leading to a slowdown the API’s molecular mobility. To recognize whether or not antiplasticization is indeed a key mechanism responsible for physical stability improvement of EZT, the prepared binary drug-polymer systems were measured utilizing differential scanning calorimetry (DSC). At the beginning of these studies, freshly obtained systems were heated up from 298 K to 453 K with a 10 K/min rate. Figure 5A presents the thermograms registered during these experiments. As can be seen, the DSC traces of all systems reveal a characteristic broad endothermal event located in the vicinity of 320–380 K, which is associated with the water evaporation absorbed by the polymer. Since the signal from water evaporation covers the area where *T_g_s* of the systems should be present, based on the data presented in panel A of Figure 5, it is impossible to assess whether or not an employed polymer exerts an antiplasticization effect on EZT. To solve this problem, samples have been dried for 5 min at 353 K, and after the drying procedure, they were heated up from 298 to 453 K with a rate of 10 K/min. The time and the conditions of drying were selected in a way that did not affect the recrystallization tendency of the tested systems. Results obtained from these studies are presented in panel B of Figure 5. It can be clearly seen that the dry systems, except for EZT-PVP/VA 5%, are characterized by a single glass transition event that moves toward higher temperatures with increasing PVP/VA content. This result proves that the employed polymer indeed works as an antyplasticizer of EZT. It is worth highlighting that the obtained results are consistent with the data of other, previously investigated systems containing EZT and low molecular weight or polymeric stabilizer [39]. Consequently, a general conclusion can be made that molecular mobility is a key factor responsible for recrystallization in the amorphous form of EZT. Thus, each procedure slowing down this pharmaceutical’s molecular mobility should lead to the effective improvement of its physical stability. Returning to the described above thermograms, one can note that two of the tested systems (i.e., those with 5% and 20% of PVP/VA) recrystallized during further heating. The onset of the exothermal process related to the drug recrystallization was registered at 370 K and 400 K for EZT-PVP/VA 5% and EZT-PVP/VA 20%, respectively. The shift in the crystallization temperature proves that EZT’s physical stability increases with increasing PVP/VA content. Finally, in the temperature range from 420 K to 440 K, on the DSC thermograms of the systems in which the recrystallization process was registered, the melting of the crystalline fraction of EZT is visible as an endothermal event.

From a simple comparison of the enthalpies of crystallizations and fusions, it can be concluded that: (i) EZT-PVP/VA 5% from the beginning was only partially amorphous, which is in good agreement with the XRD data, and (ii) in the case of the EZT-PVP/VA 20% system, only a small part of EZT recrystallized during the performed DSC experiment.

### 3.4. Wettability Study

The wetting of powders is a prerequisite for several processes important from the perspective of pharmaceutical sciences, including dissolution, disintegration, and solubilization [43]. Surface chemistry and energetics govern the interactions with water or other media, which is particularly important from the perspective of BCS class II drugs such as ezetimibe. Given that the surface properties depend on the molecular structure, varying between polymorphic and amorphous forms, the assessment of the solid-liquid interfacial interactions plays a significant role.

**Figure 6:**
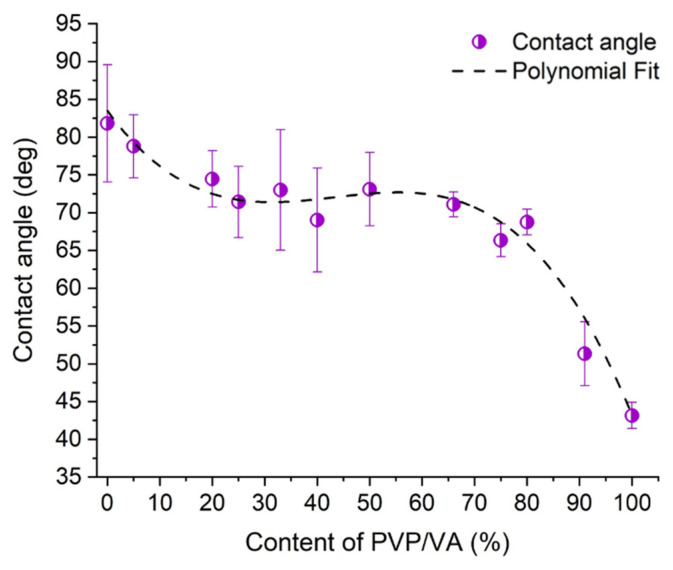
Contact angle of raw EZT and EZT-PVP/VA solid dispersions plotted as a function of the carrier concentration (circles) with fitted polynomial curve (dotted line).

The wetting behavior of raw compounds and solid dispersions was assessed by water contact angle measurements. The sessile drop technique was used to determine the hydrophobicity of the surface of prepared tablets. The measured values of contact angles were not just the proportional linear correlations of contact angles of pure compounds mixed at certain ratios (Figure 6). Such correlation was, however, observed for the samples containing more than 33% of PVP/VA, in which the differences between measured and calculated values were no bigger than three degrees. In the case of solid dispersions containing more than 40% of the polymer, the differences between experimental values and theoretically calculated averages varied between 10 and 17 degrees. Thus, we decided to find a point where the surface behavior changed. To do so, we fitted cubic polynomial y = a + B_1_·x + B_2_·x^2^ + B_3_·x^3^ to the experimental data using OriginPro 2020b software (OriginLab Corporation, Northampton, MA, USA) (Figure 6). The parameters in the equation were as follows: a = 83.51 ± 3.87, B_1_ = −0.95 ± 0.31, B_2_ = 0.02 ± 0.01, B_3_ = −1.84·10^−4^ ± 3.62·10^−5^, R2 = 0.97. The second derivative changes its sign at 43.4% of the polymer and this point was identified as an inflection point of the curve. This suggests that at the given concentration of PVP/VA in solid dispersion, the components influence the interactions of each other with water. It indicates that drug molecules interact in such a way that the surface groups of each component undergo reorganization [44]. A decrease in contact angle value in the systems containing at least 50% of the polymer may suggest that water penetrates the surface easier, which can result from the presence of the interactions between polymer and ezetimibe functional groups, such as hydrogen bonds (although no interactions were discovered using IR spectroscopy, this does not mean any interactions occured in the solution). An increase in interaction intensity between felodipine and PVP was described by Karavacs et al., who presented that strong interactions occurred for the systems exceeding 50% of polymer content [45].

### 3.5. Dissolution Study

Given the extremely low solubility of ezetimibe in water, several methods have been already applied to enhance its dissolution and yet the bioavailability. The formation of solid dispersions with either Polaxamer^®^407 or PVP K30 was described by Parmar et al. [10]. The authors applied melting and solvent evaporation techniques to obtain the systems containing 25–50% of the drug, which resulted in a 4-fold improvement in EZT dissolution. The authors did not achieve an amorphous drug; however, the crystallinity degree of EZT in solid dispersions decreased, which was concluded to affect the dissolution characteristics. PVP K30 and hydroxypropyl cellulose were also used to prepare amorphous solid dispersion nanoparticles using the supercritical antisolvent method [13]. The particles were composed of 1:9 and 2:8 drug: polymer systems and exhibited improved drug dissolution, which was inversely related to the particle size. Importantly, the formation of nanoparticles led to an increase in drug bioavailability, as the values of C_max_ and AUC_0–24_ increased by 3.2- and 2.0-fold, respectively, in comparison with EZT physically mixed with the polymers. A vinylpyrrolidone-vinyl acetate copolymer (Kollidon^®^VA64) was used as a carrier in solid dispersions obtained by hot-melt extrusion [46]. The obtained solid dispersions contained from 20% to 33% of ezetimibe and were amorphous, which was assigned to a 4-fold increase in drug dissolution.

The literature data published so far have shown only the systems containing an excessive amount of the polymeric carrier, which in some cases was still not high enough to stabilize amorphous ezetimibe. Thus, we decided to test how the addition of the different amounts of Kollidon^®^VA64 inhibits the recrystallization and improves the dissolution of EZT. Vinylpyrrolidone-vinyl acetate copolymer is widely used in pharmaceutical technology, and can act as a dry binder in direct compression, as a film-forming agent, or as a solubilizing agent. However, in ASD, it is used as an amorphous form stabilizer, which enhances the release of the API. It can be used in sustained-release formulations as a film-forming polymer but with other excipients such as polymethacrylates or cellulose derivatives [47]. It is more often used as a solubility and bioavailability enhancer especially in hot-melt extrusion [48] but also in other technologies such as spray drying [49].

**Figure 7:**
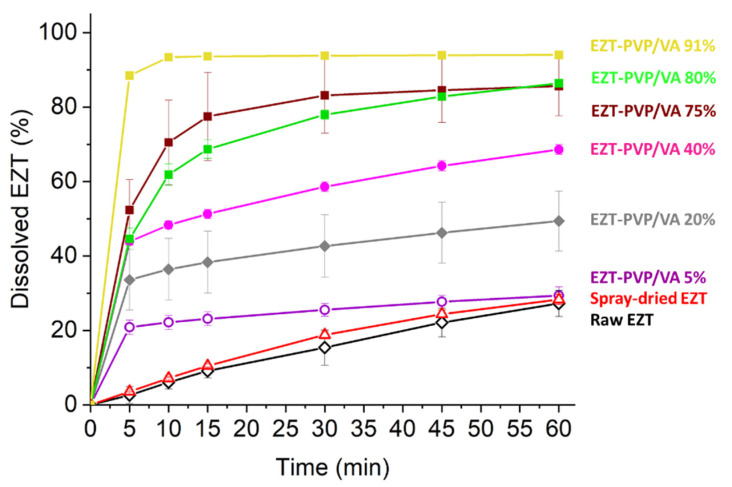
Dissolution profiles of selected EZT-PVP/VA solid dispersions, raw ezetimibe, and drug spray-dried without a carrier (content of polymer expressed as a wt%).

The dissolution profiles presented in Figure 7 showed a significant improvement in the EZT dissolution in comparison with the raw compound. In the case of a crystalline drug, 27.2 ± 3.5% dissolved after one hour. The increase in the amount of dissolved drug was not observed until the polymer was added. The amount of drug dissolved from the solid dispersion containing only 5% of the polymer was 10% higher than the sample containing crystalline powder, while in the case of the EZT-PVP/VA 91%, the amount of dissolved drug reached 95%.

**Figure 8:**
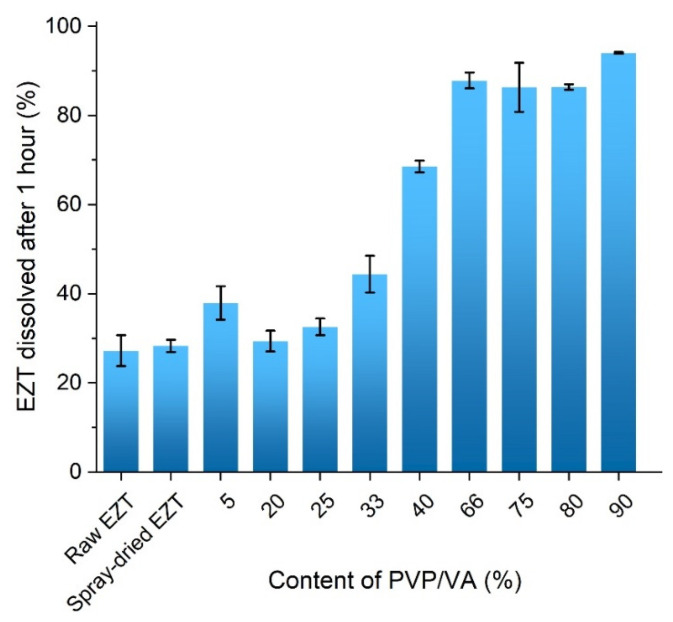
Dissolution of EZT from solid dispersions containing different amount of PVP/VA after one hour.

Interestingly, the analysis of the amount of drug dissolved after one hour of the dissolution test showed a gradual increase in the amount of dissolved drug with increasing polymer content within a range of 0–33% PVP/VA concentration (Figure 8). In the system containing 40% of PVP/VA, a noticeable increase in drug dissolution occurred, and the amount of dissolved drug reached 68.6 ± 1.3%. The second great increase in drug dissolution occurred for a system containing at least 66% of the polymer and the amount of dissolved EZT varied between 86 and 96%. The results followed the same trend noticed for the wettability results, where the contact angle plotted against the polymer content showed an inflection point at 43.4% of the polymer. This suggests that above this concentration, the system becomes independent of the PVP/VA content.

## 4. Conclusions

The present work investigated how the various content of vinylpyrrolidone-vinyl acetate copolymer in amorphous solid dispersions with ezetimibe affects the physical stability and dissolution characteristics of the drug. The diffraction patterns of the spray-dried systems indicated that even a small amount of PVP/VA in solid dispersion sufficiently stabilizes the molecularly disordered system as no Braggs peaks were present in the diffractogram. These results were consistent with the DSC data, which confirmed that the solid dispersion containing 5% of the polymer is only partially amorphous, and the increase in PVP/VA content led to an increase in glass transition temperatures, which reflects slowing down the API’s molecular mobility and can be associated with reduced crystallization tendency of the solid dispersions. The observed antiplasticization effect of PVP/VA on EZT was recognized as a mechanism responsible for the stabilization of the amorphous drug. The assumption was consistent with the lack of specific interactions between the components of the solid dispersions revealed by the infrared spectroscopy.

The amorphization of ezetimibe upon spray-drying was associated with improved drug dissolution. The dissolution data revealed that even 5% content of PVP/VA led to a dissolution improved by 10%, while the addition of 40% of the polymer resulted in the drug dissolution equal to 68%. The gradual increase in the amount of dissolved drug was observed until the polymer content reached 66%, and the amount of dissolved drug reached 90% and varied within only a few percent. This observation was consistent with the wettability determination. The contact angle plotted against the polymer content showed an inflection point around 43% of PVP/VA concentration, which indicates the point where the behavior of the system becomes independent of the amount of the polymer.

## Figures and Tables

**Table 1:** Particle size of solid dispersions obtained using the laser diffraction method.

System	D_v_(50) ± SD (µm)	Span ^1^
Raw EZT	43.7	2.7
Spray-dried EZT	91.0	4.1
EZT-PVP/VA 5%	78.9	3.3
EZT-PVP/VA 20%	34.8	3.1
EZT-PVP/VA 25%	37.0	2.3
EZT-PVP/VA 33%	36.0	2.1
EZT-PVP/VA 40%	30.4	3.5
EZT-PVP/VA 50%	27.8	4.7
EZT-PVP/VA 66%	41.6	1.7
EZT-PVP/VA 75%	51.8	1.6
EZT-PVP/VA 80%	41.8	1.8
EZT-PVP/VA 91%	31.4	2.0

^1^ Span = {D_v_(90) − D_v_(10)}/D_v_(50), where D_v_(10), D_v_(50), and D_v_(90) represent the size of 10%, 50%, and 90% of the total volume of material in the sample, respectively.

## Data Availability

Not applicable.
